# Protocol of a randomized controlled trial to test the effects of client-centered Representative Payee Services on antiretroviral therapy adherence among marginalized people living with HIV

**DOI:** 10.1186/s12889-020-09500-z

**Published:** 2020-09-23

**Authors:** Abisola Olaniyan, Stephanie L. Creasy, D. Scott Batey, Maria Mori Brooks, Catherine Maulsby, Karen Musgrove, Elizabeth Hagan, Deborah Martin, Courtenay Sashin, Christina Farmartino, Mary Hawk

**Affiliations:** 1 Department of Behavioral and Community Health Sciences, University of Pittsburgh Graduate School of Public Health, 130 De Soto Street, 6120 Public Health, Pittsburgh, PA 15261 USA; 2 Department of Social Work, College of Arts and Sciences, University of Alabama at Birmingham, Birmingham, AL USA; 3 Epidemiology Data Center, University of Pittsburgh Graduate School of Public Health, Pittsburgh, PA USA; 4 Department of Health, Behavior and Society, Bloomberg School of Public Health, Johns Hopkins University, Baltimore, MD USA; 5 Birmingham AIDS Outreach, Birmingham, AL USA; 6 Action Wellness, Philadelphia, PA USA; 7 The Open Door, Inc., Pittsburgh, PA USA

**Keywords:** Randomized controlled trial, CCRP, Representative payee, Client-centered, Harm reduction, PLWH, HIV, Antiretroviral therapy adherence, Social Security Administration

## Abstract

**Background:**

Client-Centered Representative Payee (CCRP) is an intervention modifying implementation of a current policy of the US Social Security Administration, which appoints organizations to serve as financial payees on behalf of vulnerable individuals receiving Social Security benefits. By ensuring beneficiaries’ bills are paid while supporting their self-determination, this structural intervention may mitigate the effects of economic disadvantage to improve housing and financial stability, enabling self-efficacy for health outcomes and improved antiretroviral therapy adherence. This randomized controlled trial will test the impact of CCRP on marginalized people living with HIV (PLWH). We hypothesize that helping participants to pay their rent and other bills on time will improve housing stability and decrease financial stress.

**Methods:**

PLWH (*n* = 160) receiving services at community-based organizations will be randomly assigned to the CCRP intervention or the standard of care for 12 months. Fifty additional participants will be enrolled into a non-randomized (“choice”) study allowing participant selection of the CCRP intervention or control. The primary outcome is HIV medication adherence, assessed via the CASE adherence index, viral load, and CD4 counts. Self-assessment data for ART adherence, housing instability, self-efficacy for health behaviors, financial stress, and retention in care will be collected at baseline, 3, 6, and 12 months. Viral load, CD4, and appointment adherence data will be collected at baseline, 6, 12, 18, and 24 months from medical records. Outcomes will be compared by treatment group in the randomized trial, in the non-randomized cohort, and in the combined cohort. Qualitative data will be collected from study participants, eligible non-participants, and providers to explore underlying mechanisms of adherence, subjective responses to the intervention, and implementation barriers and facilitators.

**Discussion:**

The aim of this study is to determine if CCRP improves health outcomes for vulnerable PLWH. Study outcomes may provide information about supports needed to help economically fragile PLWH improve health outcomes and ultimately improve HIV health disparities. In addition, findings may help to refine service delivery including the provision of representative payee to this often-marginalized population. This protocol was prospectively registered on May 22, 2018 with ClinicalTrials.gov (NCT03561103).

## Background

Economic disadvantage can serve as a barrier to optimizing antiretroviral (ART) adherence for people living with HIV (PLWH), driving health disparities and the HIV epidemic. Numerous studies have shown the association between financial strain and poor health outcomes including impaired functional status [1], serious health conditions [2], and all-cause mortality [3]. These associations are magnified within the HIV epidemic. Low socioeconomic resources are associated with poor engagement in HIV medical care and failed viral suppression [4], and housing instability is correlated with lower rates of engagement in HIV medical care and treatment adherence among PLWH [5]. Individuals with low socioeconomic status (SES) have higher rates of substance use and mental health issues [6–10] and higher rates of housing instability, and each of these factors is correlated with HIV health disparities [11–15]. These factors create a syndemic that produces higher rates of HIV infection; poorer engagement, retention, and adherence in care; and higher risk of negative outcomes including death [16].

It is apparent that gaps remain in interventions designed to ensure economically vulnerable individuals are engaged and retained in HIV care, representing missed opportunities to reduce HIV health disparities. Rather than focusing on these fundamental issues, most interventions seeking to improve ART adherence and rates of viral suppression work on behavioral levels, placing the burden on the individual to create change. However, behavioral changes are greatly influenced by cultural and socioeconomic factors and are, therefore, highly variable [17]. Few published studies have examined the impact of structural interventions on ART adherence [17], but structural interventions, which seek to change the context in which health is produced [18], likely present the best potential for broader reach and longer effects.

Client-Centered Representative Payee (CCRP) is a structural intervention that may mitigate the effects of economic disadvantage to improve housing and financial stability for PLWH, enabling self-efficacy for health outcomes and improved ART adherence. CCRP helps clients to consistently pay their bills, prioritizing housing needs. By making these necessary payments, which often cause stress for people with low SES, clients can focus on other aspects of their lives including increasing ART adherence. CCRP may redirect the expenditure of participants’ resources toward improved health behaviors. Shifting the focus of material and biopsychosocial resources may change the context in which health behaviors are produced, contributing to higher rates of adherence and viral suppression.

CCRP modifies implementation of a current policy of the US Social Security Administration (SSA), in which vulnerable individuals who receive public benefits are assigned to Representative Payment programs. Payees are charged with ensuring beneficiaries’ basic needs are met including housing and utilities. Once payees are assigned, the beneficiary no longer has direct access to their funds, which are instead managed by the payee. In addition to financial management, payees can support beneficiaries via budgeting education and advocacy regarding use of funds. Representative payee services have been shown to decrease homelessness and money mismanagement and to improve quality of life among people with mental health or substance use disorders [19, 20]. In some settings, representative payee has been provided to individuals with serious mental illness specifically to enforce appointment adherence, with the idea that if clients must visit the provider to gain access to funds, they will also gain access to clinical care [20, 21]. However, this approach has also been associated with clients’ experiences of coercion and reduced autonomy [20, 22].

The CCRP model modifies implementation of the traditional representative payee approach by emphasizing client decision-making and goal-setting while providing financial management services through the long-standing SSA policy. CCRP adheres to all the existing policies and procedures required by SSA including eligibility determination, ensuring beneficiaries’ basic needs are met, and reporting expenditures to SSA. There are two primary differences between CCRP and traditional representative payee services. First, strategies to engender client-centeredness and self-determination are central to the CCRP approach. Emphasizing client self-determination is an important addition to traditional representative payee services as it produces trust and strengthens the relationship between the client and the provider, improving retention in care [23–25]. Second, there are differences in the timing of initiation of services. While traditional representative payee services are often provided after beneficiaries demonstrate they are not able to manage their benefits, often through repeated cycles of homelessness and/or lost checks, CCRP seeks to disrupt this process by engaging vulnerable PLWH and their medical providers in shared decision-making about the need for payee support before they are mandated to this service.

Pilot data suggest that providing CCRP to PLWH who have low SES is feasible, acceptable to the clients, and effective in improving viral suppression. Since 2007, more than 100 homeless and unstably housed PLWH have received these services through a transitional housing program called The Open Door (TOD). TOD is a non-profit organization established in 2006 to improve clinical outcomes for homeless PLWH and is the developer of the CCRP approach. Small pilot studies demonstrate high rates of satisfaction among TOD clients [26] and suggest the intervention may help improve ART adherence [27]. The results of these studies are promising but limited by small sample size and lack of control groups.

To explore the effect of CCRP on marginalized PLWH, we designed a randomized clinical trial and a non-randomized cohort study to estimate the effect of CCRP compared to usual care on ART medication adherence, viral load, CD4 counts, financial and housing instability, financial stress, self-efficacy for health behaviors, retention in care, cost, and cost-effectiveness. This manuscript is intended to present the design and rationale for the CCRP clinical trial and the associated non-randomized cohort.

## Methods

The objectives of this study are the following:

1. Test the effect of CCRP versus standard of care on ART medication adherence and viral load among PLWH who are economically disadvantaged and unstably housed. Clinical adherence will be assessed through behavioral and biological measures including self-reported appointment adherence and viral load.
2. Test underlying mechanisms associated with CCRP that contribute to changes in ART medication adherence and viral suppression rates. Quantitative (mediation analysis) and qualitative (semi-structured interview) methods will be used to test hypothesized mediators of medication adherence and viral suppression including financial and housing instability, financial stress, self-efficacy for health behaviors, and retention in care.
3. Assess the cost and cost-effectiveness of the CCRP model. An economic analysis will be conducted to model the impact of the intervention as compared with standard of care on quality-adjusted life years as well as new infections averted.

### Study design rationale

The study team determined that the RCT design is the most appropriate method of testing the stated aims in terms of rigor, cost, and feasibility. This randomized trial was designed to enroll 160 consented individuals and will randomize (1:1) to the standard of care control group or the standard of care plus CCRP intervention group. Since the intervention may have value for all study participants, there are measures in place to provide CCRP to control arm participants once their study periods have concluded.

**Fig. 1:**
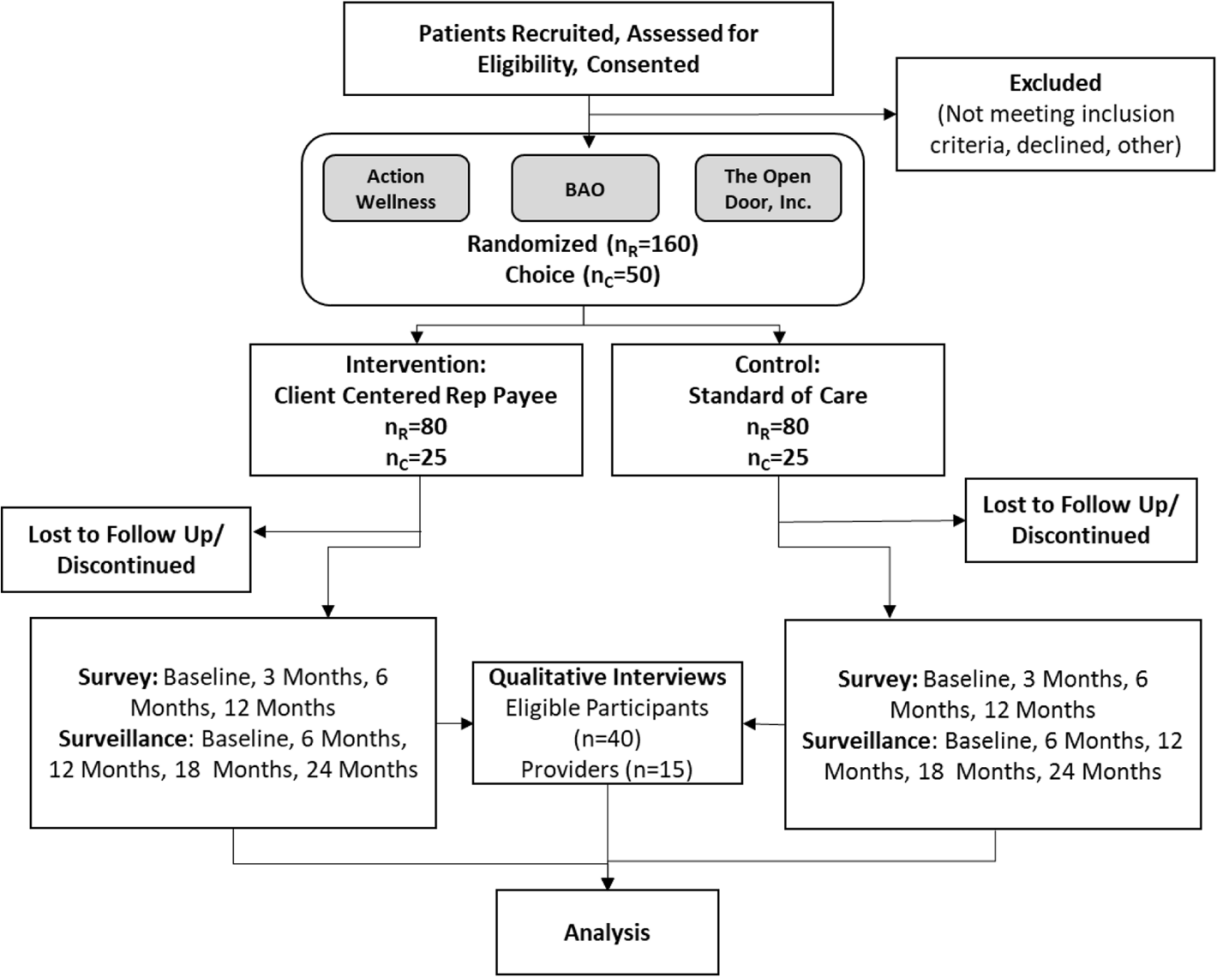
Study Design

The RCT design, however, may be a barrier for some eligible clients, specifically those who are mandated to receive representative payee services by SSA or those who experience significant health and practical challenges and require a representative payee immediately in order to stabilize their housing and health. For this reason, choice arms have been added to the study to enable observation of intervention outcomes for those individuals who are not appropriate for randomization but otherwise meet study inclusion criteria. Figure 1 depicts the study design.

### Study setting

The University of Pittsburgh will act as the study coordinating center. The study will take place in community-based organizations (CBOs) that will serve as study sites. Centering the intervention in CBOs not only allows for the intervention to build on existing, trusted relationships between participants and their case managers but also will provide an understanding of how the study operates in “real world” settings. The study sites are Action Wellness in Philadelphia, PA; Birmingham AIDS Outreach (BAO) in Birmingham, AL; and The Open Door, Inc. (TOD) in Pittsburgh, PA.

Action Wellness provides comprehensive health services to PLWH including clinical care, adherence support, supportive services, consumer education, research, and advocacy. The Open Door, Inc. is a housing first organization. The program has been serving chronically homeless PLWH since 2006 and is the organization that developed the CCRP intervention. BAO is a non-profit organization that provides case management, counseling, and legal services to PLWH. All three organizations are Ryan White CARE Act recipients.

### CCRP inclusion and exclusion criteria

The study population is limited to PLWH who are 18 years of age and older, English- or Spanish-speaking, recipients of Social Security entitlements (SSI and/or SSDI), not currently receiving representative payee services nor having received them in the past 12 months, income below 138% of the Federal Poverty Level, and one or more of the following: not virally suppressed (>200copies/ml), unsustained viral suppression over the past 12 months, or poor ART adherence. Self-report of adherence will be assessed via the CASE Adherence Index in which poor adherence is indicated by a CASE Index score of ≤10 [28]. When using the CASE Index for screening eligibility the most recent CASE score must be used and the score cannot be more than 6 months old. New clients who do not have historical viral load data but are not suppressed at baseline or who meet other inclusion criteria (poor self-reported adherence) will also be eligible for the study. Clients who have representative payees at time of recruitment or at any time during the prior 12 months will not be eligible for the study.

### Outcomes and primary comparisons

Primary outcome variables (Aim 1) include HIV medication adherence measured via viral load, appointment adherence/retention in care, and self-report using the CASE adherence index. Patients’ CD4 and viral load counts are currently collected and tracked as the standard of care by study sites, and these data will be provided to the University of Pittsburgh via data extraction from electronic health records. Appointment adherence/retention in care will be calculated via three previously validated and common ways: (a) the proportion of missed or rescheduled visits versus total scheduled visits every 6 months, (b) verification of at least one primary care visit per quarter, and; (c) two appointments in the past 12 months occurring at least 90 days apart (Health Resources and Services Administration) [29]. All data sharing will be fully Health Insurance Portability and Accountability Act (HIPAA) compliant. Additional detail regarding study variables is provided in Table 1. Some primary outcome variables will be measured via survey data, as described in Table 1. Combining biological, electronic, and self-report data on medication adherence provides the opportunity to triangulate results and obtain a more complete picture of ART adherence for study participants. Survey data will be collected at baseline, 3-, 6- and 12-month time points.

**Table 1:** Key Study Variables with Measurement Tools and Data Collection Points

CONSTRUCT	SCALE	ITEMS	DATA COLLECTION POINTS					
			**Months Since Randomization**					
**ART Adherence**			**Baseline**	**3**	**6**	**12**	**18**	**24**
HIV Biomarkers	Data Abstraction from Study Sites	CD4, Viral Load; continuous variables	✓		✓	✓	✓	✓
Sociodemographics	Self-report: HRSA reporting measures [29]	Age, race, gender, date of first diagnosis (if known), income, HIV transmission risk behavior	✓					
ART Adherence	Self-report: CASE Adherence Index [28]	3 items 1. How often do you feel that you have difficulty taking your HIV medications on time? 2. On average, how many days PER WEEK would you say that you missed at least one dose of your HIV medications? 3. When was the last time you missed at least one dose of you HIV medications?	✓	✓	✓	✓		
**Housing Instability**								
Housing Status 1	Self-report: Wolitski, et al., 2010 [30]	One item: “Which best describes your current living situation?” (Stably Housed/Unstably House/Homeless)	✓		✓	✓		
Housing Status 2	Self-report: Newly developed by team	“In the past 90 days, have you (a) Received an eviction notice or notice to vacate because your rent was not paid? (b) Had your utilities shutoff because your bill was not paid?”	✓		✓	✓		
**Self-Efficacy for Health Behaviors**								
Self-efficacy for adherence	Self-report: HIV-ASES, Johnson, 2007 [31]	12-item scale designed to assess self-efficacy for taking HIV medications	✓		✓	✓		
**Financial Stress**								
Self-report of financial stress	Self-report: Financial measures from Background Stress Inventory [32]	5 item scale: In the past month, how often did you feel distressed by the following? 1. Finding the time to pay your bills by the due date. 2. Not being able to pay your bills. 3. Unexpected events requiring additional spending that exceed your budget (e.g. vehicle repair and urgent medical attention.) 4. Existing and/or growing debt. 5. Consequences of late payments (such as having utilities shut off.)	✓	✓	✓	✓		
**Retention in Care**								
Retention in Care	Data Abstraction from Study Sites (HRSA HIV/AIDS Bureau Reporting Measure) [29]	• Proportion of missed versus total scheduled visits • Verification of at least one primary care visit per quarter • 2 kept visits separated by ≥90 days (dichotomous, ‘yes’ = retained)	✓		✓	✓		
**Additional Variables**								
Health/Mental Health Quality of Life	Single Item General Health Measure (SF-12; DeSalvo, 2006) [33]	In general, would you say your health is: (Excellent, Very good, Good, Fair, Poor)	✓		✓	✓		
Experiences of Payeeship (Intervention arms only)	Self-Report: Rosen et al., 2005 [34]	17-item questionnaire with 4 subscales: • Satisfaction with payee/case manager • Involvement of beneficiary in money management • Perceived benefit from payee arrangement • Feeling coerced			✓	✓		
Substance use	Risk Assessment Battery [35]	40-item scale assessing substance use and sexual risks	✓		✓	✓		
Depressive Symptoms	Quick Inventory of Depressive Symptomology [36]	16-item scale; self-report of depressive symptoms	✓		✓	✓		
Connections with providers	Health Care Relationship Trust Scale [37]	15-item scale assessing patient provider relationship; i.e., discussion options, committed to best care, interested in me as a person, excellent listener, accepts me, tells me complete truth, trusts me as an individual, makes me feel I am worthy of his/her time, takes time to listen, comfort talking about personal issues, feel better after seeing healthcare provider	✓		✓	✓		
Exposure to Services	Data Abstraction from Study Sites	Number of supportive services provided by Action Wellness/TOD during study period (where/how often): • Adherence support • Housing support – financial assistance • Housing support – place to stay Transportation support • Medical case management • Peer navigation • Meetings with Medical Case Manager to discuss Representative Payee	✓		✓	✓		
Social Support	MOS-SSS [38]	Five items from MOS-SSS that assess emotional, informational, and tangible functional forms of social support.	✓		✓	✓		
**Additional Variables for Economic Analysis**								
Wage level for clients	Data Abstraction from Study Sites	US Department of Labor website	✓		✓	✓		
Time spent traveling to meet with MCM regarding CCRP	Self-report: Newly developed by team	How long does it take you to travel one- way to visit with your case manager?	✓		✓	✓		
Transportation cost for participants	Self-report: Newly developed by team	How much does it cost you to travel one-way to visit with your case manager?	✓		✓	✓		
Staff personnel costs	Data Abstraction from Study Sites	Accounting records and budgets	One-time – not participant specific					
Materials and consumables	Data Abstraction from Study Sites	Accounting records and budgets	One-time – not participant specific					

The study aims involve a comparison of health outcomes between people receiving CCRP and control. The defined primary and secondary outcomes will be compared according to the intention to treat principle in the randomized trial, using a multivariable adjusted analysis in the non-randomized cohort, and using an adjusted analysis in the combined cohort of the randomized trial and the non-randomized cohort.

### Subject recruitment

Participants will be recruited during regularly scheduled visits at the study sites. In addition, staff from study sites will provide information about the study to other local providers enabling them to refer people not currently served at the sites. Flyers announcing the opportunity to participate in the study will also be posted in the waiting and patient rooms at study sites, as well as with external providers wishing to promote the study, so that individuals may self-refer. A recruitment video will be used for providers to share with protentional participants as well. Snowball sampling will be utilized in active study sites as described in section 5.4 below.

In addition, study sites will host several educational workshops to share information about the study. These sessions will take place at the offices of study sites or at community partner sites, such as other social service organizations that may refer clients to the study. Workshops may begin before the active study enrollment period and continue periodically throughout the study as needed. Need for additional workshops will be determined by study sites and the research team in response to client interest in the CCRP study. The workshops will be conducted by staff members from study sites in collaboration with the University of Pittsburgh research team. The focus of the workshops will include how the intervention works, and clients who have received CCRP will be invited to share their experiences, concerns, and perceived benefits and barriers related to CCRP services. During these workshops, no activities related to consenting, enrolling, or randomizing participants in the study will be conducted.

Qualitative interviews will begin in Year 2 of the study. Participants (*n* = 40) will be recruited by study sites from the CCRP participant pool and from eligible individuals who did not participate in the study. Participants recruited from the CCRP trial will include randomized and choice intervention and control arm participants. Since it is important to explore factors related to adherence success, qualitative interviews will include participants across all four arms who have successfully achieved viral suppression as well as those who have not. This sampling approach will enable assessment of the degree to which the intervention contributed to adherence changes, as well as mechanisms underlying change. Interviewing eligible non-participants will build understanding of why eligible participants were not interested in the study or the CCRP intervention itself. Providers (*n* = 15) will also be recruited to qualitative interviews, including those from study sites as well as from external organizations that work with CCRP participants, to explore their interpretation of their participants’ experiences with the intervention. Recruitment will occur via email and regularly scheduled staff meetings at study sites. All qualitative interviews will be voluntary, and the investigators conducting the interviews will obtain verbal consent prior to obtaining and data or feedback from participating providers. One-on-one interviews will be held in private spaces at study sites or external providers’ offices, will last 60–90 min, and will be audio recorded and professionally transcribed.

### Consent

Signed informed consent will be obtained by the research coordinators or case managers at study sites. The consent form describes study procedures, information about potential risks and benefits of participation, and contact information for further questions. Before any other study procedures take place, each participant will read and review the informed consent with study staff and given time to ask any questions they have before signing the form.

### Study participation

One hundred sixty participants will be randomized in equal proportion to the control and intervention arms with an additional 50 assigned to non-randomized choice intervention and choice control arms. All participants will be enrolled in the intervention for 12 months, with an additional 12-month follow-up period in which clinical data will be assessed. Participants who elect to participate and agree to be randomized will be assigned to the intervention or control with a 1:1 allocation ratio via a simple randomization procedure conducted by research coordinators at the study site and generated via REDCap [39]. A permuted block design was rejected in order to avoid the predictability of the intervention assignment given that the intervention is unmasked. Once participants consent to participate, a unique identifier will be generated via REDCap, which will return the assignment to the study arm. The randomization process will be monitored throughout the study period to ensure there are no systematic differences between study arms and no selection or assignment biases.

As noted previously, randomization is likely a barrier to participation for some eligible clients, specifically those who are mandated to receive representative payee services by SSA or those who experience significant health and practical challenges and require a representative payee immediately in order to stabilize their housing and health. For this reason, choice arms have been added to the study. In situations when delaying CCRP services would be unethical, individuals (*n* = 25) will be able to enter the choice intervention arm. To determine if immediate assignment to Rep Payee via the choice intervention arm is warranted, the following questions will be used to guide discussions with clients and their providers: [1] Is there risk of housing loss, as evidenced by multiple evictions, eviction notices, and/or utility shut-offs in the past year? [2] Is there risk of money mismanagement because the client is being persuaded to use money in ways they don’t want to? [3] Would it be unethical to wait to provide Rep Payee services? Additionally, 25 clients who meet study inclusion criteria will be recruited to the choice control arm to test for participant differences between arms.

Given it is readily apparent when CCRP services are provided, there is no blinding of assignment to arms for participants or study personnel. As described above, the study site will assess for exposure to representative payee services throughout the study period. At the conclusion of the study, participants who chose to be in the control group will be offered CCRP services as they may be interested in the intervention benefits.

### Intervention

CCRP will be embedded within existing services at the study sites. During the baseline visit, randomized and non-randomized intervention arm participants will also complete the SSA Request for Representative Payee Services form. SSA authorization typically occurs within 3 months. Case managers at the study sites will help the client to create a budget and prioritize expenditures, focusing on housing and utilities in order to enhance housing stability. The case manager, therefore, will become the point-person for the CCRP service. Money management will be provided by the CCRP financial manager who will set up a bank account on behalf of the client; case managers do not have access to the participants’ accounts nor will be responsible for payment of bills.

Once the SSA authorizes the organizational payee, Social Security entitlements (SSI and/or SSDI) will be sent directly to that account. Checks or electronic transfers will be paid by the financial manager directly to the billers including landlords and utility companies. The financial manager will not be identified to the client, which is a safety precaution and also helps to ensure that discussions about budgeting and practical needs become part of the ongoing conversation between the client and the case manager.

Rep Payee responsibilities include the following:

- Case managers will meet with participants on a regular basis to understand their needs and help them develop monthly budgets. Participants will work with their case managers to decide how they want their bills to be paid, how they want extra money to be distributed, and if they want to develop a savings plan.
- Decisions about bill payment occur between the case manager and the client. The financial manager, who is responsible for bill payment, will follow the plan set forth by the client in collaboration with the case manager. The financial manager will not make spending decisions that vary from this pre-determined budget.
- Study sites will ensure that participants’ funds are being used in the participants’ best interest.
- Study sites will keep detailed records of how bills are paid in order to provide an accurate report to SSA when they ask for that and will complete all accounting forms as required by SSA.
- Study sites will report events to SSA that may affect participants’ benefits, including death or incarceration.
- Study sites will follow all other rules as set by SSA. Printed copies of SSA requirements will be made available to participants throughout the study.

Throughout the course of the study, the research team will follow safeguards including those specified by the SSA to ensure the protection of participants. These safeguards are specific to oversight, keeping finances secure, preventing identity theft, paper and electronic file securing, and protecting beneficiary bank accounts.

Participants assigned to the randomized and non-randomized control arms will continue to attend visits in keeping with the study sites’ standards of care and normal operating procedures. Participants randomized to the intervention will receive the same care in addition to CCRP. Staff from study sites will assess for exposure to representative payee services throughout the study period to ensure that, if control group participants elect this service through other providers, this can be controlled in the mediation analysis. Otherwise, all other interventions are considered standard of care and no other interventions are prohibited during the study. At the conclusion of the study, participants randomized to the control group will be offered CCRP services, ensuring that all participants have the opportunity to benefit from the intervention.

#### Withdrawal from study or intervention

Participants can withdraw from the study by notifying their case managers at the study sites. Additionally, participants in the intervention groups can request to have the study sites removed as their representative payee at any time in the study. In these cases, they can also be removed from the study or remain in the study, in which case they will be considered off-protocol. If participants no longer want study sites to serve as their representative payee and feel they are able to pay their bills independently, the study sites will assist in this process. This will include advocating with the participants’ medical providers to sign off on the SSA paperwork indicating that the service is no longer needed (SSA-787 “Physician’ s/Medical Officer’s Statement of Patient’s Capability to Manage Benefits”). If the physician feels the participant still needs help from a representative payee and the participant disagrees, their case manager will work with the participant to understand why they feel they are ready to pay their bills independently. If the participant is able to demonstrate financial independence, the case manager will accompany the participant to SSA to provide a signed “third party” statement explaining that they have direct knowledge of the participant’s ability to pay bills independently. If SSA does not agree to have the study site removed as organizational representative payee, the study sites will help the participant appeal this decision. This will include linking the participant to free legal services, if needed. Study sites can also help the participant identify a different representative payee if desired by the participant.

### Analysis plans

#### Study assessments

Participants in the four arms will complete self-assessment tools at baseline, 3-, 6-, and 12-month time points. Viral load, CD4, and appointment adherence data will be monitored at baseline, 6-, and 12-months, as well as 6 and 12 months after the participant’s active study period. Since many individuals who receive ART often achieve viral suppression within 3–6 months, these time points will provide appropriate opportunities to detect shifts to viral suppression as well as persistence of viral suppression over time. Self-assessment variables are described in Table 1. During the baseline visit, intervention arm participants will also complete the SSA Request for Representative Payee.

The study employs mixed methods to explore primary outcomes and underlying mechanisms contributing to changes in adherence and viral suppression rates (Aim 2). These methods include a mediation analysis, process measures, survey measures, and qualitative interviews. The mediation analysis will test the causal chain in which it is hypothesized that adherence is improved, which includes self-efficacy for health behaviors, retention in HIV clinical care, medication adherence, CD4 counts, and HIV viral loads. It will also enable exploration of any variance in study outcomes: if adherence is not improved for all intervention participants, these variables may explain why and provide information regarding additional supports that may be needed. Process measures include number of contacts with providers, which will be extracted from records at study sites.

#### Statistical approach

Statistical analyses will be conducted by the Epidemiological Data Center at the University of Pittsburgh Graduate School of Public Health. For each of the specific aims, an initial descriptive analysis of all available data will involve summary statistics and exploratory data analysis techniques. These strategies will be used to describe the individual and combined distributions of observed variables of interest, ascertain the correlation structure among the variables, and examine the necessary assumptions for subsequent statistical techniques. If the assumptions for any proposed statistical test are not met, the data will be transformed or a nonparametric alternative will be used.

The intention-to-treat principle will be used for all primary analyses designed to compare outcomes between the intervention groups (i.e. CCRP or usual care) in the randomized clinical trial. Analyses in the non-randomized cohort will be conducted according to the group that they choose at the time of study entry.

The comparisons of the randomized and choice intervention groups for the primary outcome variables will be conducted with an alpha level of 0.05; an alpha-level of 0.01 will be used for the other outcome variables in order to adjust for multiple comparisons. For each continuous outcome measure, a linear mixed effects model will be constructed with the follow-up outcome measures as dependent variables, with the randomized and choice intervention group, follow-up time, and corresponding baseline measure as independent variables, and with a random intercept term to account for within-subject correlation. All participants with at least one outcome value will be included in the models since linear mixed models account appropriately for data missing at random (MAR). Non-linear mixed models using a binomial link will be used for binary outcome measures. The significance of the coefficient of the intervention term is the primary test of the intervention main effect. An interaction (i.e., the product term) between follow-up time and intervention group will be added to test whether the effect of the intervention differs over the follow-up course.

For comparisons that include the non-randomized “choice” participants, a propensity score will be created that estimates the likelihood of an individual person choosing the CCRP. This propensity score will be included as a co-variate in the mixed models for study outcomes to account for confounding factors. The interaction between the propensity score and treatment group will be included and tested in a subsequent model to determine if the benefits associated with the CCRP intervention differs for patients who are more likely to choose CCRP.

Participant dropout and other sources of missing data may occur despite efforts to minimize these events. We will compare the baseline characteristics and intermediate outcome values between participants with missing outcome data and those with complete outcome data. We anticipate that some patient factors will be significantly associated with missing data. We will adjust for the observed factors associated with the missing data patterns in the primary analyses and will use models that appropriately account for MAR data. If concerns about missing data remain, multiple imputation or pattern mixture models will be used to account for the observed missing data patterns.

#### Power and sample size

ART has been shown to have a dramatic effect on viral load and CD4 cell counts for PLWH [40]. Since the control group will also have access to treatment, we conservatively estimate that the observed between-group standardized effect size in this trial will be between 0.33 and 0.50 [i.e., Platten, et al., showed that treatment with ART increased CD4 cell counts from 210/μL to 410/μL, a 95% increase, and viral load was reduced (to under 50 copies /mL) in 91% of participants [40]]. Based on existing data, an effect size of 0.50 is reasonable for ART therapy in a broad population. Moreover, an effect size of 0.5 is generally considered medium [41], and we designed our trial to have power to detect modest effects for this intervention. Using a two-sided inequality hypothesis test and a two-sample t-test with alpha = 0.05, we determined the samples sizes required to provide 80 and 90% power to detect varying effect size differences between the two assigned treatment groups.

Based on these estimates, we plan to enroll a sample of 160 participants in the randomized study arm. If greater than 80% of participants contribute at least some follow-up data, the trial would have 128 participants with analyzable outcome data. This study would then have 80% power to detect a difference between the intervention and control groups of 0.5 standard deviations (SDs). For other outcomes where analyses are based on an alpha = 0.01, the study would have 80% power to detect an effect size of 0.608. Since 0.50 is considered a medium effect size, the trial is powered to detect medium effect sizes between the intervention groups for these key outcome measures.

#### Qualitative interviews

The University of Pittsburgh research team will digitally record and transcribe interviews with the study participants, eligible non-participants, and providers. A thematic analysis will be conducted in NVivo 12 [42] using contextualizing and categorizing strategies. First, the interviews will be explored for major themes to contextualize the data. Then, the research team will develop a set of analytic codes, derived from the exploration of themes as well as a priori hypotheses. All the interviews will be coded, and at least 20% of the interviews will be coded by two researchers and compared for consistency. Results will be discussed with the research team to triangulate and validate the findings.

#### Cost, cost threshold, and cost-utility analysis

The University of Pittsburgh will manage the transfer of de-identified data from the study site to researchers from Johns Hopkins Bloomberg School of Public Health, who will conduct a cost-effectiveness analysis. The cost-effectiveness analysis will use existing parameters from the literature as well as de-identified data from the study site including those collected via participant self-report (time spent by clients traveling to and from services, transportation costs to and from services, and HIV risk behavior) and data abstracted from the study site (number of participants enrolled, number of client contacts, time spent by clients in service, wage level for clients, staff personnel costs, materials and consumables costs, and viral suppression.) Data will be used to estimate the cost of delivering the intervention, the cost-effectiveness threshold, and the cost-per-QALY (quality-adjusted life-year) gained.

### Data management

Data that is abstracted from patient records at the study site will be collected by the study site and provided to the Coordinating Center via a scheduled transfer using PittBox as a temporary transfer vehicle. Data will be deleted from PittBox within 24 h of scheduled transfer and then uploaded to a Pitt department managed server. Client self-report data and tracking forms (off-protocol, events) collected through computerized tablets will be stored on Pitt department managed servers. To minimize loss of confidentiality, we will ensure that participant ID numbers are used to identify study materials. All materials with identifying information, including consent forms, will be maintained separately from the study materials, and will be secured per the study site’s security policy and approved per the local IRB.

Qualitative data will be collected by researchers from the Coordinating Center. A digital recorder with 256-bit file encryption and device PIN locking will be used to record the semi-structured interviews (*n* = 40 participants and 15 providers). The audio recording will be transferred to a Pitt desktop, transcribed, and imported into qualitative analysis software. The Pitt desktop utilizes encryption software. The audio recording will be permanently erased from the portable recorder. The study site will link abstracted and self-report data to participants recruited to the qualitative interviews so that that these data can inform the interview questions. All data are held at the study coordinating center; thus, only those involved with data analyses have access to the final trial dataset.

### Monitoring

An external Data and Safety Monitoring Board (DSMB) has been appointed, consisting of a biostatistician, an HIV physician engaged in research, and a community advocate with a strong understanding of health-related research. This committee is independent from the sponsor and competing interests. This committee will monitor the study, advise the NIH Program Office, and provide input to the PI and study team regarding data safety and quality. The Epidemiology Data Center (EDC) statisticians will provide a summary report to the PI on a regular weekly basis to enable monitoring of study recruitment and will establish a monitoring plan for study outcomes. The PI will, in turn, report to the DSMB, which will monitor accruing data, protocol deviations, and Serious Adverse Events (SAEs). The PI will convene the board at least once a year to confirm that the participants in the trial are being cared for safely.

The PI and the EDC will train the study site using the Manual of Operations to ensure that most updated versions of the study protocol are utilized and to orient staff members to the Data and Safety Monitoring Plan, including reporting responsibilities. When necessary modifications are made to the Manual of Operations, including those requiring IRB approval, an operations memo is sent to all study sites and reviewed in biweekly meetings between the study sites and PI. The team has created a system in which the study site will use an Event/Problem form to report events to the PI, who will then review to determine if the criteria have been met for Adverse Event, Serious Adverse Event, or Unanticipated Problem. Adverse events will be monitored via expedited reporting of SAEs that are unexpected and related to the study protocol to the DSMB members and study team, the scheduled reporting of adverse events and study outcome event rates to DSMB members on a semiannual basis, and the monitoring of the study outcomes by assigned intervention group on an annual basis. The DSMB may advise early termination of the trial for safety reasons or make other recommendations regarding modifications to the protocol.

### Confidentiality

To minimize loss of confidentiality, the research team will use anonymous participant ID numbers to identify study data. Only those people who participate in qualitative interviews will have their names and contact information shared with researchers from the University of Pittsburgh so that interviews can be arranged. These will be removed from all records at the University of Pittsburgh once the interview is complete. All materials with identifying information, including consent forms, will be kept in double-locked filing cabinets at the study intervention site or within a password-protected electronic database with no potential access by modem or other means.

### Patient and public involvement

There was no direct involvement of participants, participant advisors, or the public in the study design or research questions. However, the CCRP intervention was developed by TOD with intentional input from peer advocates at this grassroots organization. In addition, BAO has a committed Community Advisory Board (CAB), the support of which was sought and gained prior to study implementation in Birmingham. Additionally, this CAB has contributed to decisions about the study including those related to incentives and recruitment. Participant involvement in the study includes completion of self-assessment surveys at regular intervals as well as participation in qualitative interviews at all study sites. Results will be disseminated to participants and other PLWH through existing communication methods at study sites.

### Research ethics approval

The University of Pittsburgh Human Research Protection Office (HRPO) initially approved this study via expedited review on October 4, 2017. The IRB of the City of Philadelphia Department of Health (DoH) is not accredited by the Association for the Accreditation of Human Research Protection Programs; therefore, the University of Pittsburgh will not enter into a single IRB agreement with the Philadelphia DoH. Both review processes were required because the study site operates under the purview of the Philadelphia DoH and that entity will not cede IRB review to the University of Pittsburgh HRPO. The City of Philadelphia IRB first approved this study on April 6, 2018. With the addition of subsequent study sites in Birmingham, AL and Pittsburgh, PA, study sites in these areas (TOD, and UAB, which provides ethical oversite to research conducted at BAO) ceded to the University of Pittsburgh HRPO.

## Discussion

The primary goal of this study is to assess the degree to which CCRP helps marginalized PLWH improve their ART adherence. The study is challenging in that it aims to recruit a large number of PLWH to an intervention in which they will no longer have access to their SSA entitlements. Thus, trust in the provider-participant relationship and multiple participant safeguards are critical. An additional challenge is related to the fact that assignment to representative payee is the role of SSA, so date of onset of the intervention can be delayed due to issues beyond control of the study team. The study was initiated in 2018 at the first study site in Philadelphia, PA. However, in 2019 the inability to recruit the requisite sample at this site resulted in the termination of recruitment activities (though the study remained active for those already enrolled) and in the addition of two subsequent study sites. Recruitment was again suspended in early 2020 due to the COVID-19 pandemic in accordance with NIH notice NOT-OD-20-087. During this time, study activities continued on a remote basis for participants already enrolled.

Study outcomes may provide information about supports needed to help economically fragile PLWH improve health outcomes and ultimately improve HIV health disparities. In addition, findings may help to refine service delivery including the provision of representative payee to this often-marginalized population. While the representative payment program has been operating for decades, there is little extant literature describing best practices in this approach, including the specification of client-centered practices and policies. The economic analysis is designed to provide information regarding the cost of the intervention to participants, programs, and society. Improving engagement and retention in care for PLWH with low SES can significantly reduce the cost of care in the US, and findings from threshold analysis may provide valuable information regarding the number of quality-adjusted life years (QALY) that would need to be averted to make a claim of cost effectiveness and the number of HIV transmissions that would need to be averted to make a claim of cost savings.

Trial results will be disseminated via publications in refereed journals, presentations at scientific conferences, and communication processes currently used by the CBOs serving as study sites so that participants and other PLWH will have access to study findings. Findings may also have utility for policy makers, clinicians, and supportive service providers interested in improving adherence for PLWH who are economically fragile.

## Data Availability

Not applicable.

## Abbreviations

CCRP: Client-Centered Representative Payee
ART: Antiretroviral therapy
SSA: Social Security Administration
SSI: Supplemental Security Income
SSDI: Social Security Disability Insurance
BAO: Birmingham AIDS Outreach
TOD: The Open Door, Inc.
PLWH: People Living with HIV
